# How much detail is needed in modeling a transcranial magnetic stimulation figure-8 coil: Measurements and brain simulations

**DOI:** 10.1371/journal.pone.0178952

**Published:** 2017-06-22

**Authors:** Petar I. Petrov, Stefano Mandija, Iris E. C. Sommer, Cornelis A. T. van den Berg, Sebastiaan F. W. Neggers

**Affiliations:** 1 Dept of Psychiatry, Brain Center Rudolf Magnus, University Medical Center Utrecht, Utrecht, the Netherlands; 2 Center for Image Sciences, University Medical Center Utrecht, Utrecht, the Netherlands; 3 Department of radiotherapy, University Medical Center Utrecht, Utrecht, the Netherlands; University of Toronto, CANADA

## Abstract

**Background:**

Despite TMS wide adoption, its spatial and temporal patterns of neuronal effects are not well understood. Although progress has been made in predicting induced currents in the brain using realistic finite element models (FEM), there is little consensus on how a magnetic field of a typical TMS coil should be modeled. Empirical validation of such models is limited and subject to several limitations.

**Methods:**

We evaluate and empirically validate models of a figure-of-eight TMS coil that are commonly used in published modeling studies, of increasing complexity: simple circular coil model; coil with in-plane spiral winding turns; and finally one with stacked spiral winding turns. We will assess the electric fields induced by all 3 coil models in the motor cortex using a computer FEM model. Biot-Savart models of discretized wires were used to approximate the 3 coil models of increasing complexity. We use a tailored MR based phase mapping technique to get a full 3D validation of the incident magnetic field induced in a cylindrical phantom by our TMS coil. FEM based simulations on a meshed 3D brain model consisting of five tissues types were performed, using two orthogonal coil orientations.

**Results:**

Substantial differences in the induced currents are observed, both theoretically and empirically, between highly idealized coils and coils with correctly modeled spiral winding turns. Thickness of the coil winding turns affect minimally the induced electric field, and it does not influence the predicted activation.

**Conclusion:**

TMS coil models used in FEM simulations should include in-plane coil geometry in order to make reliable predictions of the incident field. Modeling the in-plane coil geometry is important to correctly simulate the induced electric field and to correctly make reliable predictions of neuronal activation

## Introduction

In neuroscience and medicine, Transcranial Magnetic Stimulation (TMS) is increasingly used to investigate brain function as well as for diagnostic and therapeutic purposes. During TMS administration a rapid, short-lasting magnetic field is generated which induces a relatively focal electric field in the cortex. Such externally induced electric field can cause depolarizations or hyperpolarization of the ion-channels in the cell membranes of cortical neurons, leading to alterations in neuronal activation or ultimately in neuronal plasticity. This is exploited in various ways both in research and in clinical settings to modulate human behavior, to diagnose and to treat conditions affecting the central nervous system [1].

However, the spatial and temporal pattern of the actual effect of a TMS pulse on the brain tissue is not well understood, let alone the ensuing changes in activity patterns of ensembles of neurons. The interaction of a rapidly changing magnetic field with the brain tissue is complex and depends on the exact cortical morphology, realistic tissue conductivity, and last but not least the exact geometry of the TMS coil and the current running through it.

The increasing adoption of TMS has recently inspired several groups to computationally evaluate the induced electric fields in the human brain [2,3]. A variety of computational models of TMS induced currents in the brain exists, such as Finite Element Modeling (FEM), Boundary Element Modeling (BEM) [4,5] and Impedance Methods (IM) [6]. The aforementioned studies focus on how different brain tissues, anisotropy and shape influence the induced electric fields. However, there is little consensus on the preferred method how to model the magnetic field of a typical TMS coil, which serves as an input to the just mentioned numerical methods. Approaches vary significantly from simple idealized coil models [3,6] to detailed models of realistic stacked coil winding turns [2,5]

One quite common approach towards modeling a typical TMS coil is to adopt simplified geometries in the form of idealized circular shapes (one per 'wing' in case of figure-of-8 coil). The idealized dipole models on a single layer disk [7], elliptic integration on a perfect circular contour [3] or piecewise Biot-Savart law integration over each line segment of a single circular thin wire [8] are just a few examples of such idealized coils. Others have experimented with more elaborate designs where the detailed geometrical properties of the coils are better captured. The Biot-Savart law, which provides the magnetic field around a straight wire piece, can be applied in principle to any shape of packed coil winding turns. It was applied on a thin wire with elliptic geometry by [9], while [5] [10] additionally incorporated wire width, wire height and number of turns to model even more geometrically realistic coils. By improving the idealized dipole model of [7], a more detailed and better shaped dipole model was then proposed and adopted by Thielscher and colleagues [11,12] [2].

Although results from these studies show realistic currents patterns in the crowns and lips of the cortical gyri, they often lack direct empirical validation of the adopted coil models. This makes further predictions of ensuring neuronal activation rather problematic, since the reported results directly depend on the accuracy of the magnetic field predictions.

One exception is the work presented by Salinas et. al. [5]. They have not only looked at the discrepancy between simplistic and detailed coil geometries, for four commercially available coils, but also compared results against empirical measurements. Those measurements were conducted using field pickup coil probes and an oscilloscope, measured at a couple of control points. Their results show that differences between simplistic and detailed coil models diminish at distances of 3 cm or more away from the coil, while the biggest discrepancy of 32% can be observed close to the coil surface (< 2cm). They concluded that coil model details have a minor impact when TMS is applied on humans, but it might be rather significant when applied on small animals. They also highlight the need to further determine and evaluate the complete electric field.

An alternative approach to modeling a TMS coil is suggested by [13] [14]. The proposed method of measuring, mapping and storing the magnetic vector potential in a data base circumvents the need of modeling the coil all together. The need of inner design knowledge for each coil type and manufacturer vanishes too. It can also be used for validating purposes in place of the MR imaging methods adopted in our study. One obvious advantage of our method is its non-invasive nature that would be beneficial in case of future in-vivo experiments with human subjects.

A previously demonstrated, MR measurements [15] [16] can be utilized to reconstruct the TMS magnetic field from the acquired phase maps [17]. Those studies serve as proof of concept, but lack either a realistic stimulator or/and coil, which is a major limitation when it comes to validation of TMS coil models (see section Material and Methods → Theoretical Background). We have developed a novel setup to allowing successful application of both a real TMS stimulator and TMS coil inside a clinical 3T MR scanner [18].

We consider three distinct models to represent a typical figure-of-8 TMS coil. We start with the arguably the most often adopted in common literature model, the single loop of thin wire with the outer most radius for each wing. Then we gradually introduce more geometrical detail to such an idealized model to better match it’s geometry to the shape of the physical coil. The second model consist of several spiral winding turns and the third one has a few stacked layers of the same spiral windings. We adopt BiotSavart integration to numerically predict the induced magnetic field of each model and we compare it against MR field measurements. The focus of our study is not to find the most accurate model for a given TMS coil but rather to assess the validity of geometric approximation used in published TMS literature, most notably the very simplistic in our opinion coil, where each wing is modeled using only a single wire/winding turn.

To estimate the relevance of detailed TMS coil modeling for actual brain stimulation, we extrapolate the predictions of the three coil models to the human brain. In particular, we focus our attention on a small cortical patch in the motor cortex near M1 (the 'hand knob' area) using FEM. We devise a simple metric to quantify potential differences in prediction of cortical excitation for each of the coil models. These simulations should allow one to relate our findings on appropriate TMS coil models to actual brain stimulation experiments, as one will have a simple scalar 'activation' measure per coil model to compare between coil models.

This exploration can offer guidance for realistic TMS induced current simulations in a human brain that are increasingly suggested to have the potential to improve TMS treatment planning [19,20]. Unraveling the influence of the TMS coil model may help to bring TMS models into clinical practice.

## Materials and methods

In the sections below we present the 3 different coil models of increasing geometric complexity that we investigate, the most detailed being close to an actual MR safe TMS figure-of-8 coil. Next, we will describe how the Biot-Savart method is implemented and proceed with the empirical validation of the predicted fields by comparing the simulated against the measured TMS induced magnetic field. Finally we describe simulations of the electric fields induced by the coil models on a realistic human brain model. This will allow us to access the impact of each model in a more realistic context where we look to quantify the potential of neuronal activation.

We investigated three different figure-of-8 coil models of increasing complexity. Coil BSM-811 is the most trivial of all, a single layer with two circular loops of a single wire with opposite currents running in each. Then coil BSM-819 is a more elaborated coil, a single layer of nine spiral winding turns per coil wing. Finally, the most complex coil BSM-879 constitutes of seven layers each having nine spiral winding turns per coil wing. Here, BSM stands for Biot-Savart-Method and 8 refers to the general geometric shape of what we refer to as a typical figure-of-8 TMS coil. See the results section ‘MRI field measurements’ for a graphical overview of the three coil models.

We developed in-house a few additional modules to SCIRun 4.7 (A Scientific Computing Problem Solving Environment (Scientific Computing and Imaging Institute (SCI), Utah, USA)). The following modules were introduced to generate the geometry for each model: Modules→TMS→ModelTMSCoilSimple for coil model BSM-811; Modules→TMS→ModelTMSCoilSpiral for coil models BSM-819 and BSM-879. The underlying algorithm for both modules is unified, implemented as half circle generator of points (mesh nodes) in the range 0-π with angular step equal to the range / #elements (number of segments Table 1) at offset R from the origin. The spiral shape of BSM-819/879 was realized simply as half-circles with planar (x-axis) offset of the origin and radius, R_n_ + dr/2 = R_n+1_ and O_n_ + dr/2 = O_n+1_ where dr = (Router—R_inner_ / # winding turns) and n is winding index. The amount of current in the wire is provided explicitly and assigned on each element of the wire mesh (segment) as a scalar value [+/-]. To compute numerically the induced magnetic fields in accordance to the Biot-Savart formulation (see Eqs 2 and 3) we introduced one final module to SCIRun, Modules→Math→SolveBiotSavartContour, when provided with a SCIRun mesh of type 'curve-mesh' it iterates over each segment and accumulate the contribution on each to the final field in discrete steps interpolated along n to n+1 nodes (see Table 1 integration step). It treats negative sign for the current as a hint to reverse the direction of integration on each segment, causing a flip in interpolation from n+1 to n. This last addition conveniently helps in composing wire segments independent of topology (order of segments/nodes in the mesh) thus making the job of the generator-modules more trivial.

**Table 1:** Geometrical details of the three modeled coils.

Coil Type	Segments Per Wing	Integration Step	Radius Innermost Winding	Radius Outtermost Winding	Outer Interwings Distance	#Layers	#Winding turns
BSM-811	92	0.025 mm	44 mm	44 mm	2 mm	1	1
BSM-819	763	0.012 mm	26 mm	44 mm	2 mm	1	9
BSM-879	5341	0.04 mm	26 mm	44 mm	2 mm	7	9

Overview of the three coil models, geometrical dimensions and additional modeling details.

The source code for the additional modules that we developed in-house for this study is available online at: https://github.com/pip010/scirun4plus/releases/tag/v4.7.2 with DOI 10.5281/zenodo.160114

### Theoretical background

The E-field produced by a TMS machine has a primary and secondary component[3]. The primary component $\vec{E_{p}}$ arise from the TMS coil magnetic vector potential $\vec{A}$, which solely dependent on the coil geometry, inductance and pulse shape. The secondary component $\vec{E_{s}}$ is related to the gradient of the scalar potential of the volumetric conductive medium Φ, which contains the portion of the electric field actually linked to the resistive brain tissues. This latter electric field can be related to charge accumulation at tissue boundaries caused by gradients in the electric tissue conductivity. Under the quasi-static approximation the total electric field $\vec{E_{t}}$ induced by TMS is given in Eq 1.

$$\vec{E_{t}}=\vec{E_{p}}+\vec{E_{s}}=-\frac{\partial \vec{A}}{\partial t}-\nabla \Phi$$ (1)

To numerically evaluate the magnetic vector potential we used the Biot-Savart formulation, which gives the magnetic field distribution around a current flowing through a wire segment dl at a distance r-r_0_ away, where r >> dl.

$$A\left(\vec{r}\right)=\frac{I\mu_{0}w}{4\pi }\int \frac{\partial l}{\left|\vec{r}-\vec{r_{0}}\right|}$$ (2)

Here w is a scalar weighting factor specific for each coil model (reported in Table 2) and μ_0_ is magnetic permeability of free space at a distance r from the source. The total vector potential field is used as input to our FEM simulation to derive the approximate solution for $\vec{E_{s}}$ in Eq 1 (see section 'Human Brain Simulation” subsection 'Finite Element Simulation').

**Table 2:** Coil current values used in simulation.

Coil Type	Phantom Experiment (1% MO)	Human FEM Model (70% MO)	Current Distribution Factor (w)
**BSM-811**	25032 [mA]	288.75 E10^6^ [A]	* 7
**BSM-819**	3576 [mA]	38.5 E10^6^ [A]	* 1
**BSM-879**	511 [mA]	5.5 E10^6^ [A]	/ 7

Currents used for each coil model for both empirical phantom experiments and the human FEM simulation

The piecewise Biot-Savart method we adopt to compute the magnetic field of our coil models is as follows:

$$B\left(\vec{r}\right)=\frac{I\mu_{0}w}{4\pi }\int \frac{\partial l\times \vec{r}}{{\left|\vec{r}-\vec{r_{0}}\right|}^{3}}$$ (3)

Eq 3 was used to compute the magnetic field for all three coil models. Only the magnetic field was considered for validation purpose (see section 'empirical validation of coil models'.

The integral in Eqs 2 and 3 was approximated via step summation over discrete line segments, what we refer to as integration step is the uniform length taken along each segment. In order to eliminate any significant variation due to numerical computational inaccuracy we performed a tuning procedure to determine the maximal accepted integration step. The magnetic field is well known anywhere along the mid-line passing through the center of perfectly shaped circular wire according to the following analytical formula:

$$\vec{B}=\frac{I\mu_{0}}{2\sqrt{{\left(R^{2}/Z^{2}+R^{2}\right)}^{3}}},$$

where I is the delivered current, R is the radius of the circular coil, Z is an offset along its central/middle axis. For a single circular coil of radius 44mm composed of 64 segments we kept the error within 1% at a distance of 1 cm from its center. The adopted integration step for each of the three coil models is reported in Table 1.

The TMS stimulator we used in the validation experiments produces a short 0.4 ms bipolar pulse (2.5 KHz). For 100% MO (machine output), the peak current and voltage amounts to 5500 A and 1650 V respectively, as reported by manufacturer.

Since the readings of our experimental setup are based on MR phase accumulation images and the result will depend on the reconstruction procedure it is important to clarify key aspects first. The recordings are MR phase images in the interval +/- *π* per pixel. Those raw images were then post-processsed through unwrapping algorithm. The resulting phase patterns represent the net (time average over the TMS pulse) MR phase contribution. In theory, if the bipolar TMS current running in the coil would have the same amplitude and duration for both the current polarities, the total MR phase contribution would be zero. In practice, however, since the current running in the TMS coil is a damped bipolar pulse, the phase contribution given by the first current polarity is not fully compensated by the phase contribution of the second current polarity, thus leading to a measurable MR signal. In principle, the same phase contribution can be given by a static DC current running in the TMS coil for the same duration as the actual bipolar TMS current. We call this DC current as equivalent TMS current.

**Fig 1:**
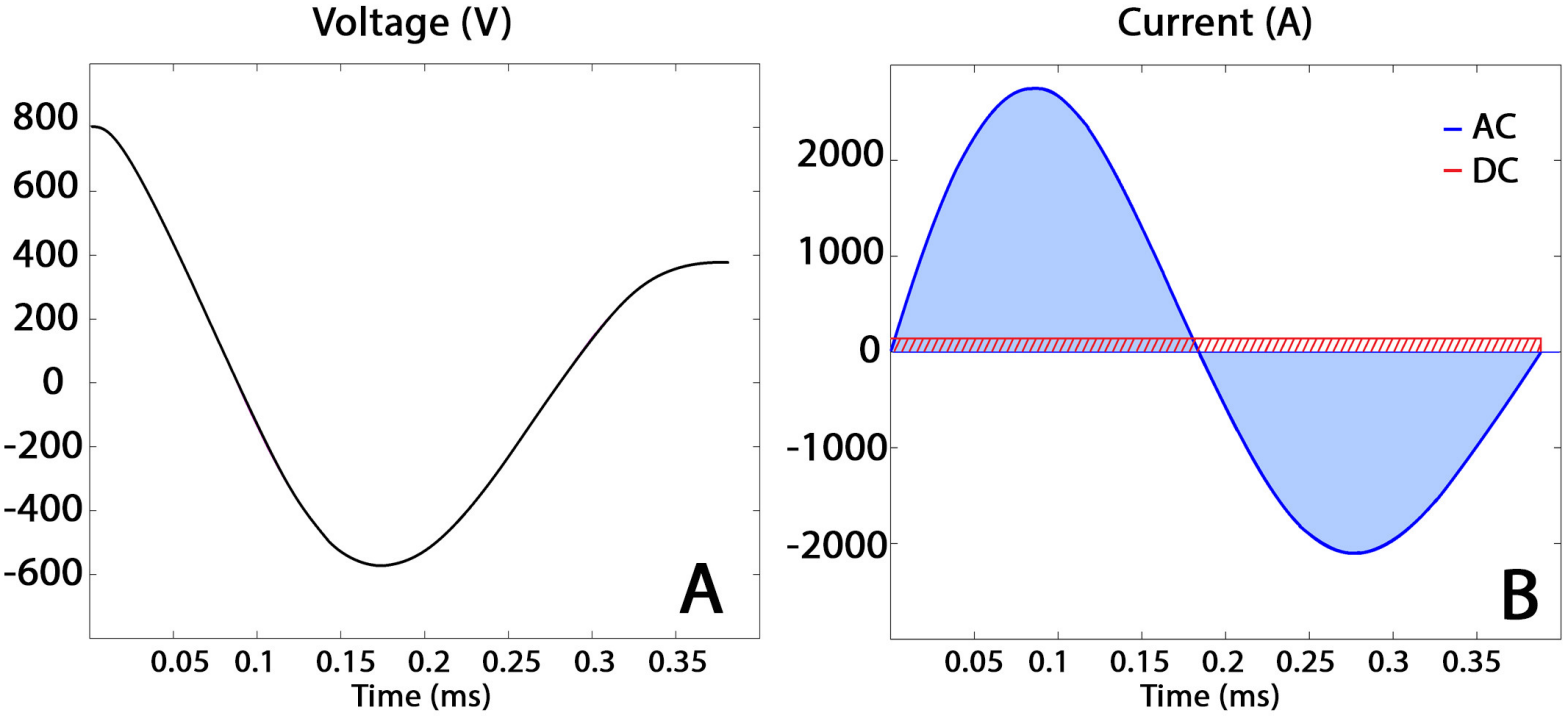
Coil current approximation. On the left (Fig 1A) is shown the plot of the electric field of the coil, while on the right (Fig 1B) it is shown the resulting current profile. The DC approximation in red (shaded area) Fig 1B. Both subplots are idealized and given for 50% machine power.

To approximate the TMS pulse to its equivalent DC current, we calculated the time averaged integral of the current shape normalized to 1% MO, see Fig 1B. For each coil model, the obtained values of DC current used to compute both the incident magnetic field and magnetic vector potential is reported in Table 2. We choose 1% MO to avoid image artifacts (signal loss due to excessive intra-voxel dephasing) near the coil during the MRI measurements [18].

The current distribution factor is a compensation factor to the net current provided for each model to account for any discrepancy due to pure geometrical differences between the three coil models. For example coil BSM-819 is considered closest to the real geometry of the actual physical coil, nine winding turns of thin wire for each wing, where the current for coil BSM-811 is 7 times the one for BSM-819, roughly equal to the ratio in the wire length between the two models. Finally, for coil BSM-879 the current is split equally among each of the seven layers.

To compute the secondary electric field induced in brain tissues during a TMS experiment, i.e. the field produced by the charge accumulation at tissue boundaries, the input to drive the FEM simulations should be the maximum dI/dt. This ratio refers to the first half frame of the current shape in Fig 1B. Considering a typical MO for TMS experiments of 70%, the maximum dI/dt in our case will be 38.5x10^6^ A/s. This value is in line with reported in literature values for 100% MO [21,22].

### Empirical validation of the coil models

In order to empirically validate the three coil models, we performed MR-based measurements. First, we introduce the apparatus adopted for the experiment. Then, we describe the MR acquisition protocol and data processing.

#### Apparatus

Experimental measurements were conducted inside a 3T MR scanner (Achieva, Philips Medical Systems, Best, The Netherlands). For TMS administration, we used the Magstim Rapid 2 TMS stimulator (MagStim, Whitland, UK) connected to an MR compatible TMS coil with ceramic casing. For all details of this setup see a previous report from our group [23]. Measurements were conducted on an agar phantom (diameter 12.5 cm, height 20 cm filled with a solution of Agar 20 gr/L and NaCl 9.5 gr/L: conductivity 1.6 S/m at room temperature of 23°C and frequency 128 MHz). This phantom was placed into a custom built holder that allowed additional positioning of the TMS coil and MR elliptical surface coils (flex-L and flex-M) for signal reception.

To make the TMS coil visible in the acquired images, twelve additional markers filled with tap water were fixed on the posterior coil surface.

#### MRI acquisition and data analysis

**Fig 2:**
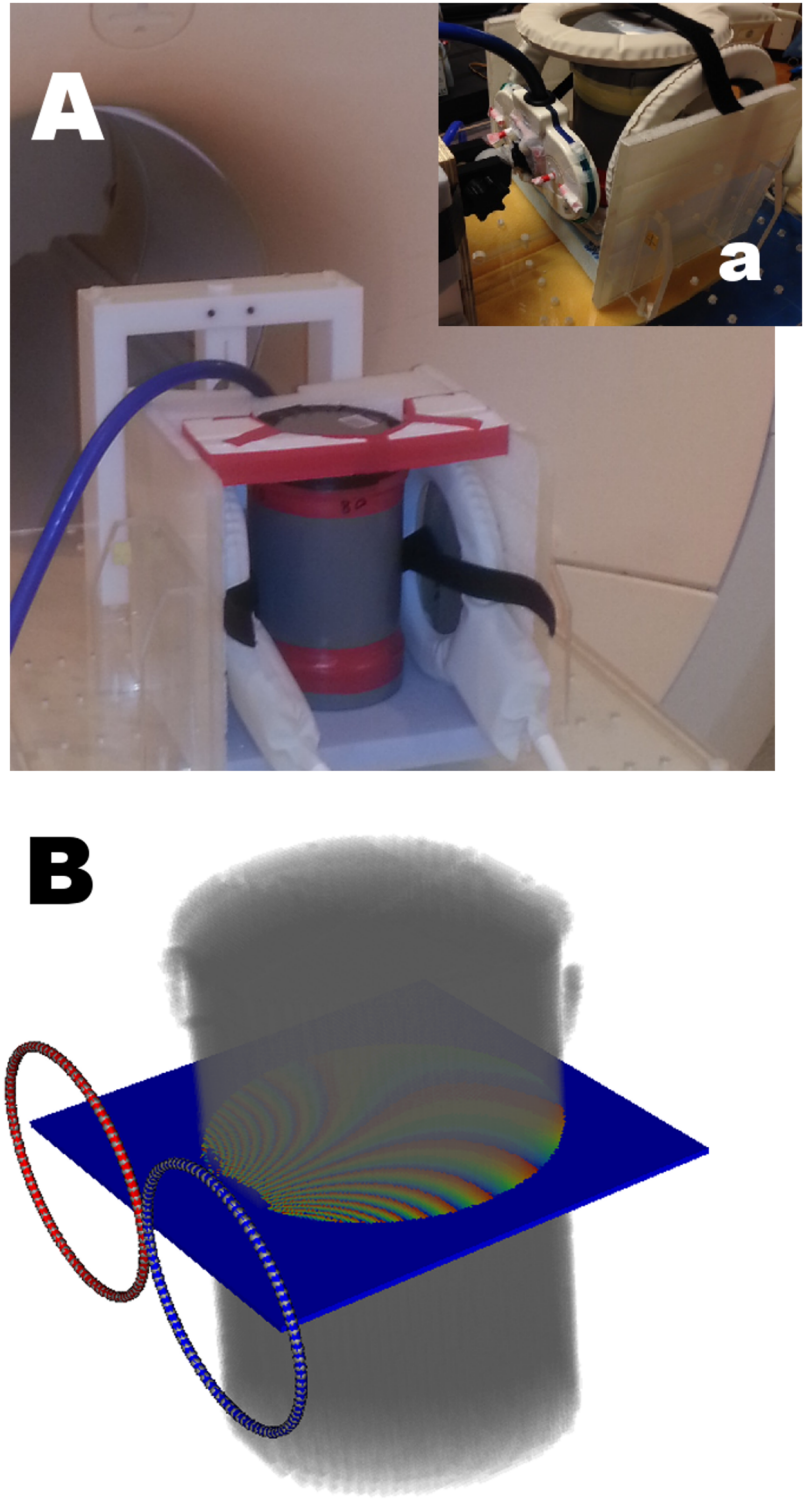
Experimental and virtual setup. On top (Fig 2A) photo of physical phantom; holder; MR flex coils. Fig 2Aa, top right, the actual TMS figure-of-8 coil with capsules visible in red. On bottom (Fig 2B) is shown a visualization of the phantom T2, coil model BSM-811 as well as a single slice of MR phase accumulation measurements.

For the purpose of TMS magnetic field mapping, a single echo Spin Echo sequence was performed, using the body coil in transmit and the MR-flex coils in receive mode. For this measurement the parameters were: repetition time TR = 1 s, echo time TE = 20 ms, field of view FOV = 160x160x2 mm^3^, voxel resolution RES = 1x1x2 mm^3^. The relative position of the TMS coil with respect to the phantom is depicted in Fig 2.

By subtracting two phase images acquired with and without applying TMS pulses, it is possible to isolate the TMS contribution to the phase accumulation [18]. Due to the direct relationship between phase accumulation and the incident TMS magnetic field[15], it was possible to retrieve the TMS incident magnetic field. However, these maps reflect only the z-component of the total magnetic field. This is because, in an MR experiment, only the magnetic field component parallel to the main static magnetic field B0 is measurable.

To reconstruct in simulation the position of the TMS coil with respect to the phantom, a reference T2 weighted turbo spin echo map of the phantom and the TMS coil was acquired: TR = 11 s, TE = 80 ms, FOV = 240x240x210 mm^3^, and RES = 1.5x1.5x3 mm^3^. Then we used a commercially available stereotactic navigation system “The Neural Navigator” (www.neuralnavigator.com, by Brain Science Tools BV, The Netherlands) to co-register the coil position and orientation from MR world space to simulation world space. The process involves capturing the position of several fluid capsules affixed on the coil casing, using 3D digitizing hardware. Those positions were then mapped to the location of the visible capsules in T2 MRI space via point cloud algorithm [24]. We achieved 1 mm precision for position and up to 4 degree precision for orientation. To compensate for the thickness of the coil case, a rigid body translation of 15 mm was applied as the last step. Further details on the coil position reconstruction method and precision can be found in an earlier paper of our group using the same method [23].

### Human brain simulation

This study directly validates several models of increasing geometrical detail and complexity of the figure-of-8 TMS coil using MR techniques. However, to allow readers to interpret the differences between coil models for their actual brain stimulation experiments, we need to evaluate the electric field evoked by each model and how it interacts with brain regions of interest. In order to assess the consequences of TMS coil model detail for use in planning actual brain stimulation, we used a finite element model (FEM) of the human head. We estimated the electric field flux through a small region in the motor cortex. Finally, we adopt a crude metric for evoked brain stimulation, that takes into account individual cortical folding patterns.

This simulation is relevant for real TMS applications, as so called motor evoked potentials (MEPs), an electromyographic recording of the motor cortex response coming from the thumb muscle shortly after a TMS pulse, are commonly measured to asses motor cortical excitability in different forms and shapes. MEPs are known to be altered in several diseases affecting the central nervous system and investigated for potential diagnostic use. For an overview see [25].

This extrapolation of our findings to the human brain will allow researchers and computational modelers to get an idea how TMS coil model detail affects predictions in realistic situations, which in the future could allow for more accurate dosimetry. Below, we evaluate the simulation of the induced activation for the thumb area in the human motor cortex (M1).

#### Human head model

We used a 3D tetrahedral mesh of a real human head, that was previously reported in literature [26], to explore the expected effect of TMS. The mesh consists of 480,316 nodes and 2,785,034 elements. Generally the more nodes a model has the higher the numerical accuracy is and the more elements a model has the better the representation of the underlying structure is. The brain mesh is partitioned in 5 compartments: Scalp, Skull, CSF (Cerebral Spinal Fluid), GM (Cortical Gray Matter), WM (Cortical White Matter). The following isotropic conductivity values were adopted for each tissue type: Scalp = 0.5 S/m; Skull = 0.02 S/m; CSF = 1.6 S/m; GM = 0.3 S/m and WM = 0.25 S/m, within average of reported values [21].

**Fig 3:**
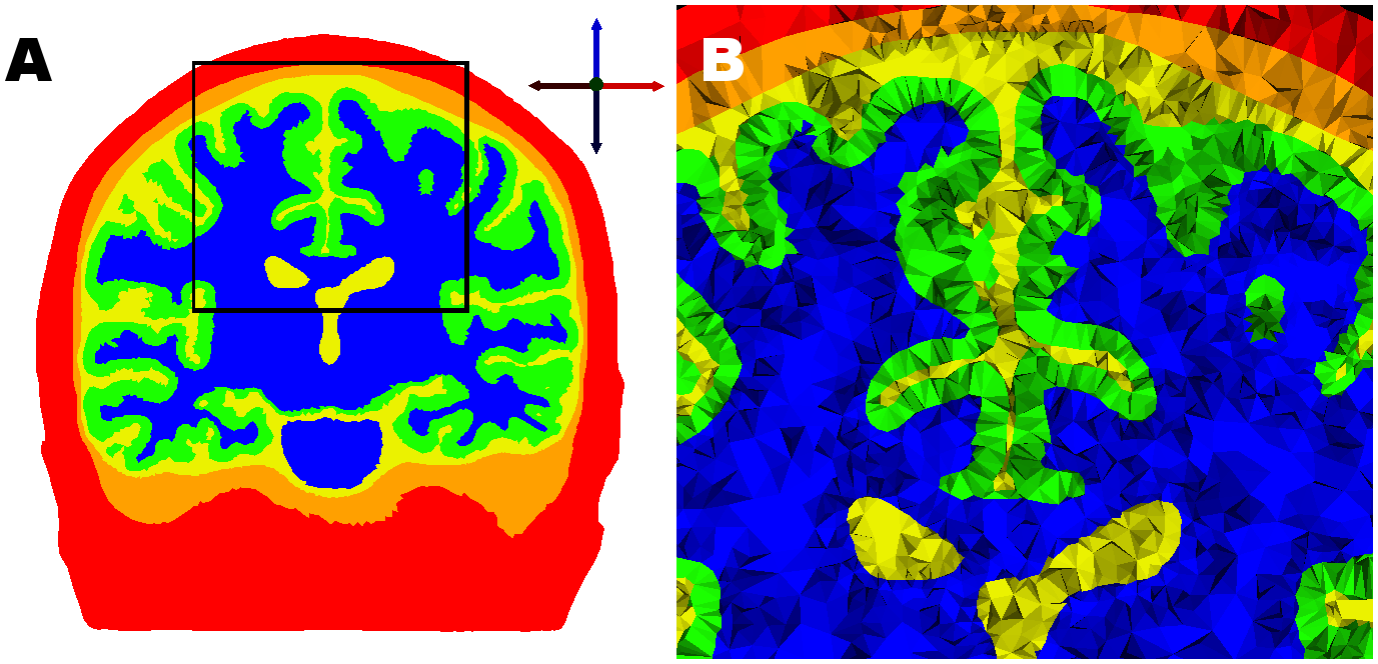
Volumetric tetrahedral mesh of the human head (FEM) model. On the left (Fig 3A) a mid-coronal slice with well conforming to anatomy boundaries for each tissue. On the right (Fig 3B) a closer view of 3A, the black sided rectangle in Fig 3A, where individual pyramidal shapes for each tetrahedron are easy to discern.

The CSF ↔ GM boundary surface is the most significant interface to consider when trying to evaluate the cortical effects of TMS [27]. The head model we employ has a relatively high quality GM outer surface with well conforming anatomically shape, see Fig 3.

#### Finite element simulation

The FEM calculations were carried out using version 4.7 of SCIRun: A Scientific Computing Problem Solving Environment (Scientific Computing and Imaging Institute (SCI), Utah, USA).

We used the SCIRun Math→SolveLinearSystem module with Jacobi pre-conditioner and the gradient bi-conjugate algorithm selected as solver with terminating residual target error RMS (Root Mean Square) set to 10^−4^.

Two boundary conditions and one global requirement were imposed:

- The Neumann's boundary condition $\vec{J}\cdot \vec{n}=0$ so no current leaves the head;
- The induced current density is continuous and obey flow property throughout the domain $\vec{J_{1}}\cdot \vec{n_{1}}=\vec{J_{2}}\cdot \vec{n_{2}}$
- In the quasi-static limit the divergence of the current density to be zero $\nabla \cdot \vec{J}=0$

Here J denotes the current density through a boundary element surface (triangle) having a normal n.

The solution of the FEM solver was the scalar potentials distribution. The gradient of the scalar potential produces the secondary in accordance with Eq 1. Summed together with the time varying contribution of the magnetic vector potential it produces the total electric field Et from Eq 1.

#### Cortical region of interest (ROI)

To assess 'activation' in the brain resulting from TMS induced currents, we choose a region of interest (ROI) around M1 in the so called 'hand knob', the area in the motor cortex controlling the thumb. This region was manually drawn using MRICron (http://www.nitrc.org/projects/mricron/). The binary mask containing the ROI was fitted from the 1mm isotropic voxel grid onto the polygonal mesh building gray matter in our tetrahedral head model. Since we decided to focus only on the CSF ↔ GM boundary interface the procedure results in a small polygonal patch consisting of ~500 triangle faces, spanning an area of ~4.5 cm^2^. See results section ‘cortical stimulation’ for the ROI rendered on top of the gray matter. The final value of the E-field for each polygon on the patch were extracted via linear interpolation from the tetrahedral mesh.

We decided to explore two orthogonal orientations of the TMS coil with respect to the central sulcus around M1, as it is known that MEPs are depending on the direction of induced current (which roughly runs parallel to the coil handle) with respect to the underlying central sulcus orientation [28]. An orientation parallel and orthogonal to the central sulcus near M1 were chosen to maximize the effect of coil orientation (responses are expected to be smallest for parallel orientations, see [1] for an overview). Our motivation for picking two orientation is not to study the effect of coil-orientation in general but rather to eliminate the coil orientation factor when drawing conclusions to our results.

The geometrical center of the surface of the modeled coils was positioned at a distance of 2 cm away from the GM surface. Besides visual inspection no additional aid was used for coil guidance and placement.

#### Cortical evaluation metric

We also take into account how the electric field induced by TMS interacts with neurons in the cortical layers, in a simplified scheme, and compare a metric (see below) of the resulting net electric field through the 'hand knob' between the two aforementioned orthogonal coil orientations for the 3 coil models.

As it is generally assumed [1] that pyramidal cells with their axons oriented orthogonally to the cortical layers are the main responders to TMS induced currents. We assumed that currents orthogonal to the cortical layers have a maximal effect, and currents parallel to it a minimal effect on an infinitesimally small surface patch. We hence devised a simple but physiologically plausible metric to evaluate the difference in potential for neuronal activation for each of the proposed coil models.

$$\vec{E_{c}}=\sum_{i=0}^{M}S_{i}\left|\vec{Et_{i}}\cdot \vec{n_{i}}\right|$$ (4)

The metric given in Eq 4 accounts for the angle between the total electric field Et and the normal n on each surface triangle of the patch (M number of triangles) weighted by its area S. Eq 4 will effectively favor electric field vectors perpendicular to the gray matter surface rather than parallel ones. Such an approach is motivated by the anatomical structure of the cortical layers, where axons of pyramidal neurons, running mostly perpendicular to the pial surface are assumed to pick up most of the induced current induced in the cortex [1]. Similar metric was proposed previously by Fox and colleagues [29], the cortical column cosine (C^3^) model, that claims to be able to estimate effective stimulating electric field for TMS [30] [31]. However, in addition we normalize the electric field by the are for each triangle to capture the electric field flux through the patch.

Importantly, we want to clarify that the purpose of using our formulation of the C^3^ metric as given in (4) is not to construct the best model for local electric activation of neuronal tissue by induced E-fields in all possible detail, or to validate such a model. The rather crude C^3^ metric does not reflect details regarding electro-physiological processes on the cell membrane, pre- and post-synaptic hyper- and depolarization, inter- and intro cortical layer connections etc. Work adequately modeling the interaction of $\vec{B}$ and $\vec{E}$ fields at this microscopic level is published elsewhere Rahman et al [32], for an overview, see De Berker et al [33]. Still, there is ample evidence that metrics like C3 approximate macroscopic TMS evoked activation quite well at the neuronal level [29–31] as well as at the EMG and behavioral level Kammer et al [34].

## Results

### MRI field measurements

**Fig 4:**
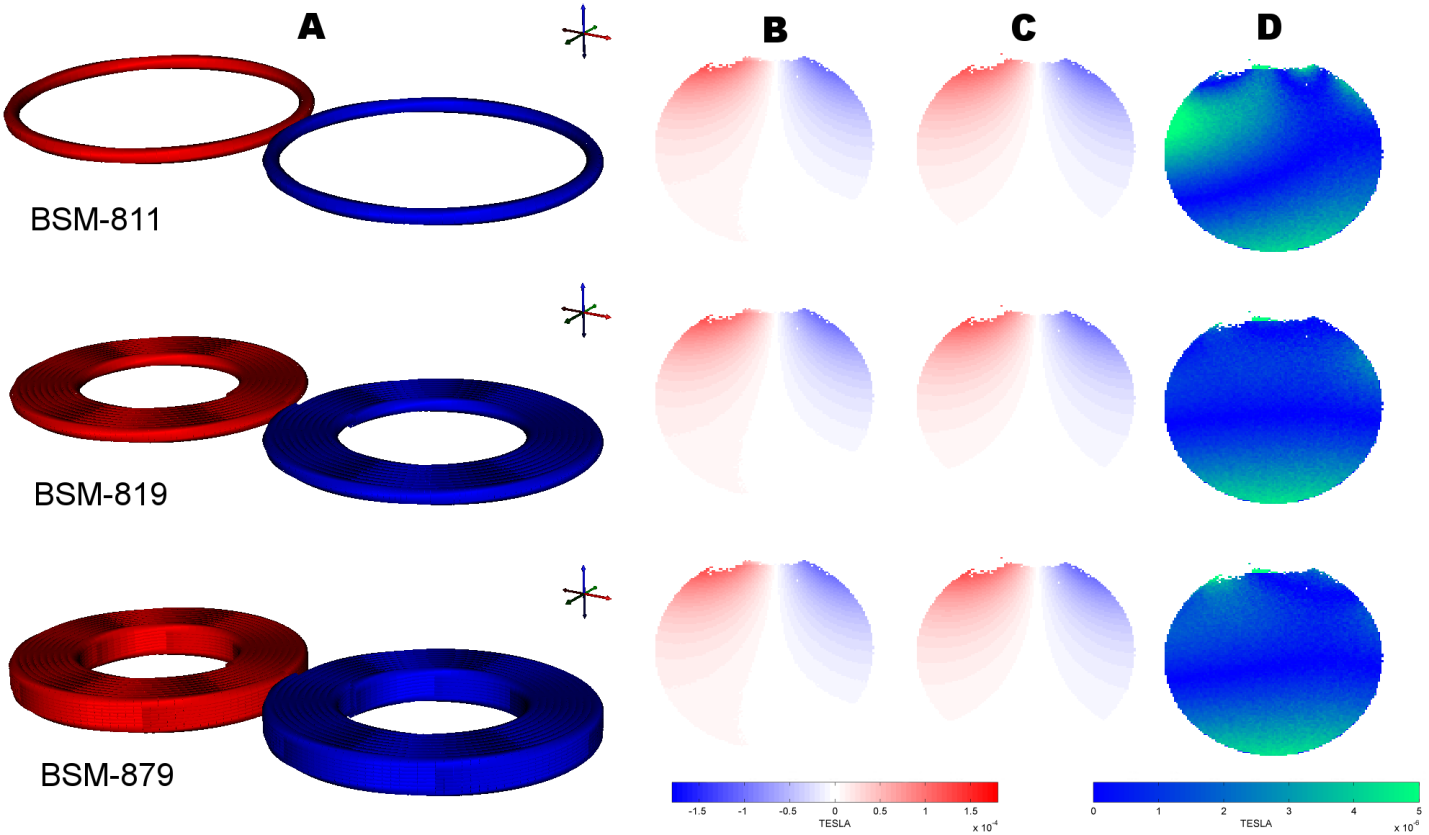
The three coil models and the empirical results. From top to bottom BSM-811, BSM-819 and BSM-879: Fig 4A the 3D models of the three coils under investigation. Fig 4B shows the Bz results, coming from MR measurements (all slices are the same). Fig 4C shows the Bz results, coming from computer simulations. Finally, Fig 4D gives the AD (absolute difference) metric for each coil model, between MR measurements (Fig 4B) and numeric calculations (Fig 4C). The slice views (Fig 4B, C and D) correspond to the 1mm thick slice depicted in Fig 2B.

From Fig 4B one can observe the raw Bz measurements from the scanner and compare it to each of the 3 coil models. Note that Bz refers to the z component of the full magnetic vector field **B**. In the same image we provide the absolute difference $AD=\left|B_{z}^{MRI}-B_{z}^{FEM}\right|$ of the same Bz field, between all coil models and the reference MRI measurement. For a distance of more than 8 cm away from the coil, the noise level becomes dominant. This is due to the low 1% machine power we employ for the empirical experiments.

Even at such low machine power and 1 mm in-plane scan resolution some signal is lost at 2cm or closer from the coil center (top view of slices in Fig 4B). Such signal drop-out is due to over-phasing of more than +/-Pi. This effectively increased the distance at which we were able to measure from 2.0 to 2.4–2.5 cm away from the coil. Nevertheless some spatial discrepancy between the simple, idealized BSM-811 coil and the more detailed BSM-819 and BSM-879 are still visible and easy to distinguish. Clear regions of over and under estimation of the simulated Bz for the BSM-811 exists in contrast to BSM-819 which has consistent error distribution (smooth gradient of the AD error metric in Fig 4), while BSM-879 pattern was still better than BSM-811 do exhibit slight inconsistency compared to BSM-819. The relative error is hardly spatially consistent, it roughly equated to about 4–10% for BSM-811 and 1–5% for BSM-819.

**Fig 5:**
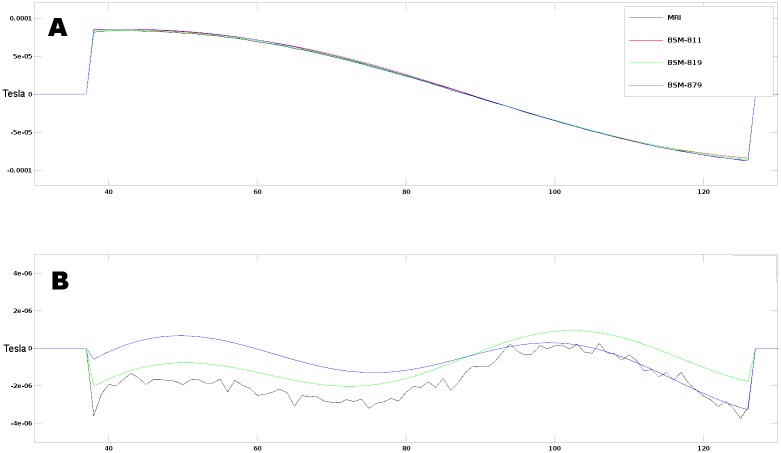
Plot of Bz field at distance 4cm away from the coil. On top Fig 5A absolute value of the measured magnetic field in the MRI and the predicted values for all three coil models. On bottom Fig. 5B The value of BSM-811 used as a baseline; MRI measures, BSM-819 and BSM-879 prediction relative to the baseline.

Overall the difference between the three coils, Bz measurement, seems quite small judging from the graphs in Fig 5, that gives us the 1D profile along the dotted line in Fig 4, situated at a distance of 4cm away from the coil. Nevertheless, the trend that BSM-811 deviates most from measurements remains the same.

### Cortical stimulation

**Fig 6:**
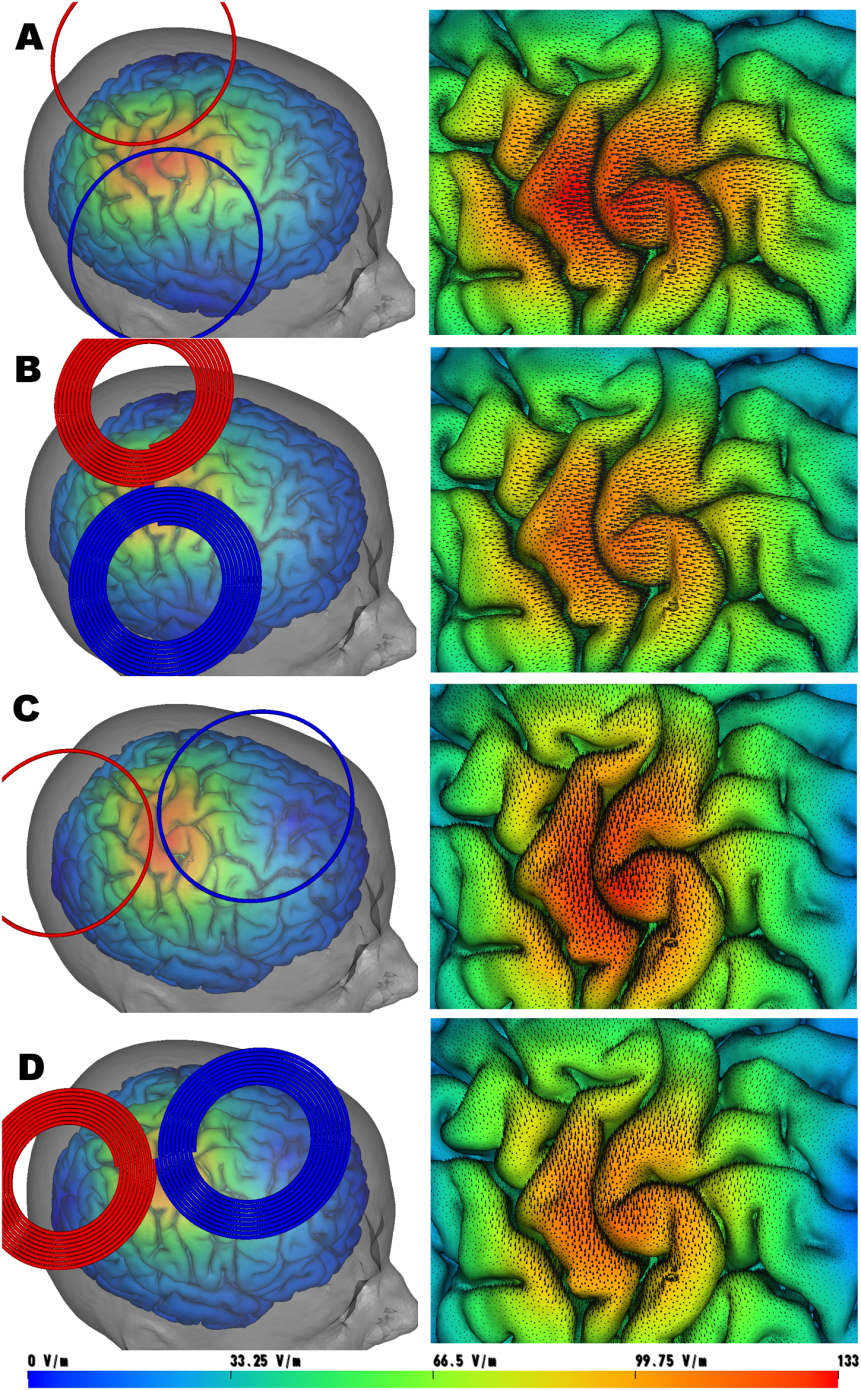
Total electric field results from FEM. On the left an overview image of the coil, the cortical gray matter sheet, semi-transparent skin rendering. On the right a close-up view (zoom-in) of the area just under the coil (M1 moto-cortex gyrus). Fig 6A coil model BSM-811 orthogonal to the M1 gyrus. Fig 6B coil model BSM-819 orthogonal to the M1 gyrus. Fig 6C coil model BSM-811 parallel to the M1 gyrus. Fig 6D coil model BSM-819 parallel to the M1 gyrus.

In Fig 6 the cortical surface and induced E-field is shown for the simple coil BSM-811 and coil with realistic spiral winding turns BSM-819, for the direction of induced current parallel and orthogonal to the central sulcus.

**Fig 7:**
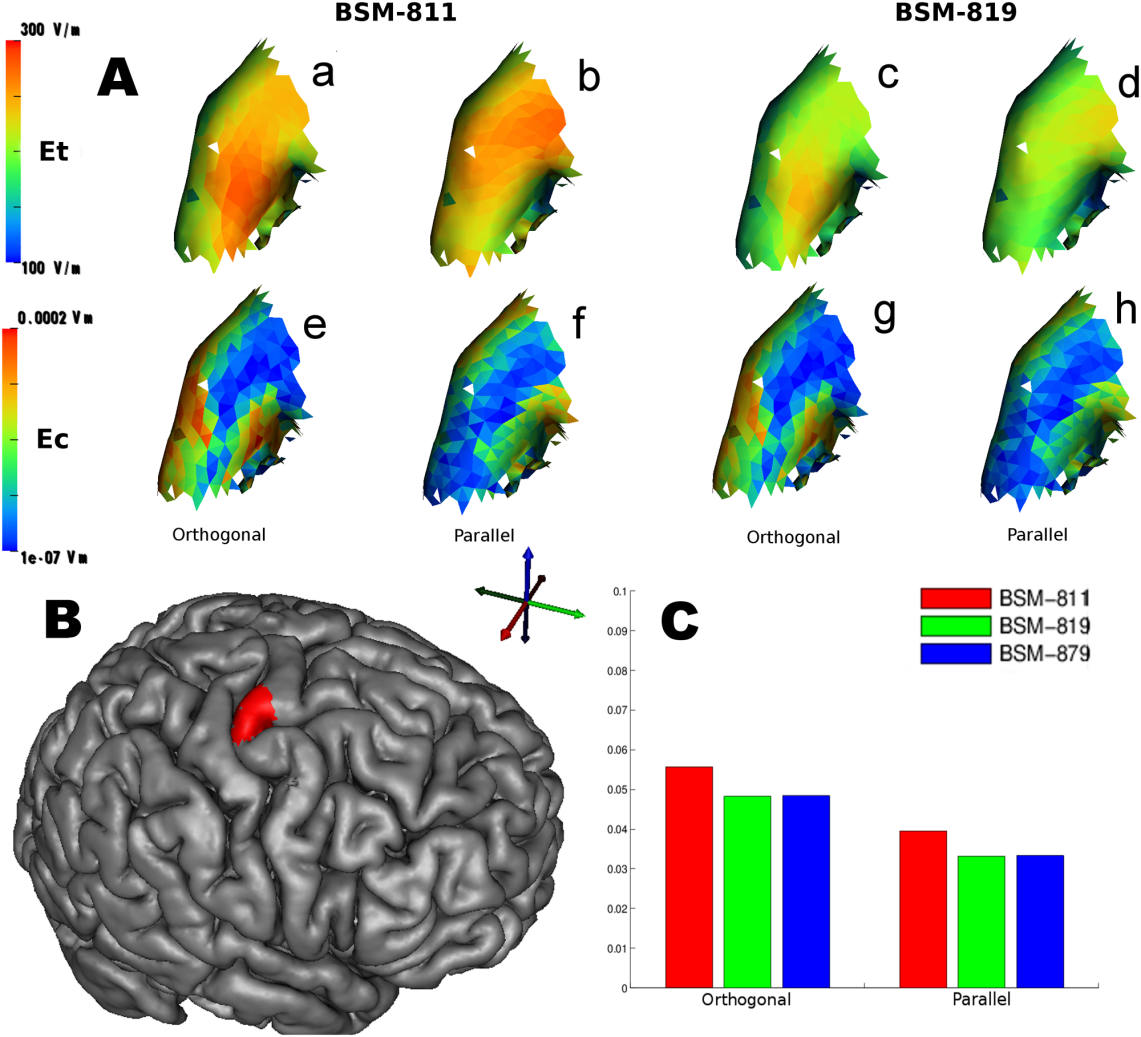
Results for the electric field(s) inside our ROI.

The three models produce visually similar shape and magnitude of the total electric field Et (Eq 1). Only the single circular loop coil has a clear overestimation of the peak area under the coil, while the results from the detailed (spiral geometry) models are indistinguishable from each other. Those observation are in accordance with the results from the empirical experiment conducted on the phantom at distance of ~3cm and evaluated on the Bz field. The discrepancy between the predicted Et field of (BSM-811) and (BSM-819) in the 'hot-spot' area under the coil is further amplified (15–20% Relative Difference) at a distance of ~2cm from the coil, see Et on the cortical GM surface Fig 6 and Et on the small ROI patch (M1 hand knob area) Fig 7.

More interestingly, however, once we carry Et to our custom cortical evaluation metric Ec (Eq 4) we can see a relative difference of around 15% between the simple and more complex coil geometries in Fig 7. The relative difference between BSM-811 and BSM-819 are 12.8% and 15.4% for the orthogonal and parallel orientation respectively. The results for BSM-879 are almost identical to the ones for BSM-819. The observed 15% relative difference between the simplified and the more complex geometries is consistent among both orientations. The relative difference of the reported metric for the two different orientations is around 33%, results are in accordance with other studies [19,35].

On top (Fig 7A) shows the total electric field (Et) and custom electric field (Ec) metrics on our ROI patch for the simple coil BSM-811 and the spiral coil BSM-819 for the two primary orientations (parallel and orthogonal to M1 gyrus surface).

Fig 7A top row (abcd) is showing the total electric field $\vec{E_{t}}$ and bottom row (efgh) is our custom electric field metric $\vec{E_{c}}$. On (Fig 7A) left half (abef) is for coil BSM-811 while the right half (cdgh) is for coil BSM-819. The two orientations are shown interleaved for each column of Fig 7A: (ae) is orthogonal; (bf) is parallel; (cg) is orthogonal; (dh) is parallel. On bottom left (Fig 7B) Gray Matter Cortex surface rendering with ROI patch colored in red. On bottom right (Fig 7C) bar-plot of our custom electric field metric $\vec{E_{c}}$ for all three coils and the two orientations.

## Discussion

In this study, we compared simulations to MR measurements of the magnetic field produced by a realistic figure-of-8 TMS coil using a real TMS stimulator and pulse shape. Three different coil models with increasing geometric complexity were considered. The 3 coil models were a simple circular pair of coils consisting of one winding, a spiraling wire per coil 'wing' with realistic dimensions and a coil model consisting of spiraling stacked wires to emulate the thickness of the wire packs. We observed that in the reagion where neurostimulation usually takes place (about 2.5 cm below the coil center), both spiraling wire coil models best predicted the actual field (RE < 5%). Instead the single circular wire coil deviated from MR measurements up to 10% RE. The difference in prediction quality between the thin and the stacked spiraling coil models was negligible.

One of the main challenges we faced was due to intrinsic limitations of the hardware we used to conduct the empirical work we presented so far. In particular, our ambition to position the coil as close as possible to the phantom while maintaining realistic machine power output were in conflict. The strong magnetic field produced just under the coil focal point causes signal dephasing that results in image loss. The effect is voxel-size dependent. Therefore, an increase of the imaging resolution can reduce the extend of the region where signal loss is observed, however at the cost of significantly longer scan time [18].

Furthermore, by using computational modeling we estimated what the net induced E-field of these 3 coil models would be on a patch of motor cortex of a typical brain, corresponding to the 'thumb area', using two orthogonal coil orientations and FEM simulations of a detailed volumetric description of brain tissues. The metric to compute 'neurostimulation' was chosen such that it reflects properties of neurons in the cortical gray matter sheet: the E-field angle with respect to the cortical surface was taken into account such that perpendicular fields lead to maximal stimulation. It was observed that both spiraling coil models had yielded a value of this net field measure that was nearly identical, whereas the idealized circular coil model deviated significantly. Also, realistic effects of TMS coil angle with respect to central sulcus could be reproduced.

We acknowledge the fact that our approach towards modeling a realistic TMS coil, which is characterized by having more complex geometry, can be further improved by incorporating more elaborated current distribution schemes [7] [12]. Instead, we decided to split equally the current between each winding of the spiral coil as well as each layer of the stacked spiral coil. While this had no influence on the results from our empirical magnetic field measurements it might had underestimated the focality of these two coils in the reported results from the numerical electric field calculations.

Our results help to predict and optimize TMS effects quantitatively, before an actual stimulus is delivered. Given the increasingly important place of TMS in clinical practice [36], such models are needed to accurately deliver TMS induced currents in the desired brain region at the desired dose. Currently, few guidance exists in how detailed the computational model of the TMS coil itself needs to be. Our results clearly demonstrate that a significantly different outcome is achieved when increasing coil detail is taken into account.

The results presented here are among the very few reports of empirical validation of a realistic figure-of-8 coil used for TMS of the human brain. Although the particular coil we investigated is specifically designed to withstand large magnetic fields and comply with MR safety protocols, the only substantial difference we observed from other figure-of-8 coils reported in literature were the ceramic filled casing and the slightly more densely packed wires. Neither of these preclude cross-comparison of other coils to our empirical field measurements.

Similarly to the work by Salinas and colleagues [5], our analysis of the results from the magnetic field measurement demonstrated that the coil geometrical details play minor role at distances further than 3 cm away from it. Unlike Salinas and colleagues, we observed that the surface area of the coil is the dominant geometrical feature contributing to the discrepancy between simplified (idealistic) and detailed (realistic) models. Salinas and colleagues, however, suggested that wire height (coil depth) rather than wire width (winding turns) is the key differentiating factor. This can be explained by differences in methodology, e.g. our 1x1 mm planar field measurement versus the sampled regularly 5mm hotspot pickup-coil measurements, or the fact they evaluate the E-field while we measure B-field only. It has been suggested [5] that the full electric field, together with secondary effects [10], needs to be considered first before drawing any conclusions in the context of human TMS. We did so using FEM simulation on a realistic human head model with a coil at a distance of 2cm away from the GM cortex. The discrepancy between the simple circular coil versus the detailed spiral coil were exaggerated further by numerical derivation of the complete E-field.

In most previous related TMS studies, when adopting the simplistic circular loop approach towards modeling a figure-of-8 shaped coil, researchers have opted for a variant of Eqs 2 and 3 where the current through the coil is weighted N times, where N is the number of winding turns. Such an approach additionally contributes to the perceived difference between simplistic and detailed models. In our particular case using 9 (for the number of winding turns) instead of 7 (the ratio in wire length) would have resulted in additional ~23% relative error in approximating the amount of current running through single circular loop coils.

Peres and colleagues [16] also attempted to map the magnetic field of a realistic TMS coil inside an MRI bore, as reported in a conference proceeding. Although that abstract shows that in principle it is possible to map the induced magnetic field with an MR scanner, they did not compare their measurements with a model to assess the validity of both measurements and model. Furthermore, an important limitation of their work is that they were not able, due to technical limitations of their setup, to stimulate with the actual TMS stimulator, but used batteries with direct current (DC) instead. It is therefore hard to evaluate how valid their observations are for estimating the induced field by real TMS coils attached to a real TMS stimulator. With our approach [18], it is possible to stimulate the TMS coil inside a 3T MRI scanner using a real TMS stimulator and a realistic pulse shape, albeit only at low intensities. Although MR phase mapping of a TMS coil is not entirely new, using a real stimulator instead of a battery or other artificial source, also tests assumptions about the temporal characteristics: the assumption that the net DC current under our model biphasic pulse shape is equivalent to induced phase difference is also validated at the same time. In theory discrepancies could have arisen here due to dynamic pulse shape fluctuations might lead to deviating phases, but obviously this did not play a big role.

Our results from the empirical coil validation indicated that at least a coil geometry using spiral winding turns should be used to accurately approximate the induced B-field of a typical TMS coil. However, TMS users generally aim to influence a specific brain area, mostly limited to a structural feature pf the cortical surface such as a gyrus or sulcus. For this reason we investigated the effect of TMS on neuronal activation in the motor cortex as EMG measurements from the associated muscles can be used to estimate the amount of activation that is fed into the cortico-spinal tract after a TMS pulse [37]. This way, a TMS user can more easily evaluate the consequences of coil models for predictions in a specific area of the brain that is well investigated, rather than a larger area below the coil. From FEM simulations we observed that the more detailed coils (the two models taking into account spiraling wires) yielded equivalent 'activation', whereas the idealized coil deviated significantly with 15% relative difference. The metric used to approximate 'activation' is based on a simplified scheme of how the total electric fields interact with pyramid cells in the cortical layers, where the axons perpendicular to the cortical surface are activated maximally for aligned electric fields. For an in-depth review motivating such a scheme, see [1] and [29]. We assessed the validity of this metric by comparing two coil orientations: one with a current induced perpendicularly and another one parallel in respect to the pre-central sulcus. We could generate strong 'activations' for perpendicular coil orientations, and weaker activation for parallel orientations, similar to the findings of neurophysiological experiments [28] and FEM based neurocomputational studies [35]. This finding provide extra confidence in the metric we employed to evaluate neuronal activation, whereas we are aware of the limitations of such a simplified scheme that does not take into account the full complexity of the layers of connected neurons in the cortex.

## Conclusions

When modeling a typical figure-of-8 TMS coil the use of an idealized outermost circular contour for each wing was found to be inadequate to accurately compute the total electric field, at a distance from the TMS coil relevant for stimulation of cortical neurons. Instead incorporating realistic wire winding turns resulted in better match to measurements. Both the predicted spatial distribution and magnitude of the field were most accurate in the case where we accounted for the surface area occupied by the spiraling coil wires. To a much lesser extent the wire height and coil thickness were contributing to the magnetic field induced by the coil. The FEM based brain simulations yielded similar results.

Thus, in order to make accurate predictions for the currents induced by TMS in the human brain we not only need to use realistic head properties, but also realistic models of the TMS coil. These models should at least account for the in-plane geometry of the coil, such as the spiraling wires of typical figure-of-8 TMS coils. Such approaches canimprove real-time neuronavigation, taking both individual tissue properties and specific TMS coil models into account. This would allow the operator not only to plan injected current with more spatial detail and in individualized patient models, but also gain a certain amount of control over the injected current dose. Current practices are crude and thus unreliable, such as the determination of the 'motor threshold' method [38]. Once achieved, TMS treatment efficacy will improve and the confidence in neurocognitive findings inferred from TMS studies will increase, helping TMS protocols to become more reliable and with less variability between individuals.
